# Prenatal diagnosis of absent right with persistent left superior vena cava: a case series

**DOI:** 10.1007/s00246-025-03951-0

**Published:** 2025-07-12

**Authors:** Stephen Worrall, Rahul Mital, James Strainic

**Affiliations:** Rainbow Babies & Children’s Hospital, Cleveland, USA

**Keywords:** Absent right with PLSVC, Fetal echocardiography, Congenital heart defect, Systemic venous anomalies

## Abstract

Persistence of a left superior vena cava (PLSVC) is the most common thoracic venous anomaly, seen in 0.3–0.5% of patients. In rare cases (0.07–0.13% of patients), however, it is associated with the absence of the right superior vena cava (RSVC), a condition known as isolated PLSVC, or IPLSVC (Kusaka et al. in JA Clin Rep, 2015; Bartram et al. in Am J Cardiol 80(2):175–183, 1997). Absent right with PLSVC itself is usually not hemodynamically significant; its discovery, however, has significant implications, specifically for future cardiac inventions such as cardiac catheterization and pacemaker placement. It is also associated with other pathologies such as coarctation of the aorta, right sided aortic arch, valvular abnormalities, and arrhythmias secondary to coronary sinus dilation (Kusaka et al. in JA Clin Rep, 2015; Bartram et al. in Am J Cardiol 80(2):175–183, 1997). For this reason, awareness of this anatomy and its variations remains critically important. It was traditionally found on autopsy or during the time of invasive cardiac procedures; but with advances in fetal echocardiography, it has become more common to be found prenatally, with an opportunity for follow-up to monitor for any complications and associated findings (Guarnier et al. in Pediatr Cardiol27(5):646–648, 2006; Kahramanoglu et al. Pediatr Cardiol 45(2):377–384, 2024). In the following series, we present the cases of seven individuals with maternal fetal echocardiographic evidence of absent right with PLSVC and associated findings on postnatal evaluation.

## Introduction

One of the most common thoracic venous anomalies is persistent left superior vena cava (PLSVC), found in 0.3–0.5% of patients. In rare cases, the right SVC may also be absent, a rare condition known as isolated persistent left superior vena cava (IPLSVC) [1, 2]. IPLSVC, first described in 1862, only occurs in 0.07–0.13% of patients and was frequently discovered as an incidental finding during a cardiac catheterization, operation, or even on autopsy [1, 2]. Little is known about the various complications and associated findings seen with this rare diagnosis. Though more literature has been recently published recently regarding the diagnosis of absent right with PLSVC, only a few case reports describe the fetal echocardiographic and postnatal findings, along with additional anomalies and complications [3–6].

IPLSVC is caused by failure of closure of the left anterior cardinal vein, coupled with flow reversal through the innominate vein leading to atresia of the RSVC and persistence of the LSVC, respectively [7]. This IPLSVC will most frequently drain into the coronary sinus (90%), the left atrium, or both via an unroofed coronary sinus [7, 8] The presence of an PLSVC should be suspected when coronary sinus dilation is noted on echocardiography and can be confirmed with an abnormal three-vessel view, in which the SVC is seen left of the arterial duct and aortic arch [6].

Venous anomalies have a high association with cardiac, extracardiac, and genetic disorders [9]. LSVC, for example, has previously been associated with an eightfold increase in having another congenital heart defect and has been shown to be strongly associated with coarctation of the aorta [10]. Less is known about IPLSVC, which further supports the importance of highlighting and reporting cases to document their associated findings.

## Case series

A single-center retrospective review of fetal echocardiography performed at our institution from 2017 to 2024 was completed and noted that 7 individuals were found to have absent right with persistent left superior vena cava, with confirmation on postnatal echocardiography (Table 1).

**Table 1:** Patient list with reason for referral, gestational age of initial fetal echocardiogram, prenatal echocardiography findings, and postnatal course

Reason for referral or prenatal echo	Gestational age at fetal echocardiogram	Prenatal findings	Post-natal Course
Suspected fetal anomalies	20	PLSVC to dilated coronary sinus and absent RSVC	Omphalitis and NICU stay for sepsis rule out
Suspected fetal anomaly (twin gestation)	31	PLSVC to dilated coronary sinus and absent RSVC	Supravalvular PS, NICU stay for prematurity, IUGR, SGA
Fetal anterior abdominal wall defect	28	PLSVC to coronary sinus to dilated coronary with absent RSVC; prominent innominate vein w/retrograde flow	NICU stay for Gastroschisis, neonatal HTN, SVT, RV dilation
Suspected fetal anomaly	27	PLSVC, PACs Congenital tricuspid insufficiency	Tethered leaflet of the tricuspid valve and tricuspid insufficiency
Suspected Fetal anomaly	24	PLSVC to dilated coronary sinus and absent RSVC	None
Suspected fetal anomaly	22	PLSVC to dilated coronary sinus and absent RSVC	None
Suspected fetal anomaly	35	Apical muscular VSD, PLSVC with absent RSVC	ASD vs PFO; NICU stay for respiratory failure and FTT

Three patients in our series were discharged after routine newborn care and four required admission to the NICU for reasons ranging from omphalitis and sepsis rule out, to repair for a gastroschisis and failure to thrive. Length of stay ranged from 2 to 122 days. Several patients had concerns for coarctation or hypoplasia of the aortic arch noted on prenatal screens, but none were seen on postnatal imaging. Other cardiac anomalies were found, including supravalvular pulmonary stenosis that required balloon angioplasty, a tethered tricuspid valve with resulting regurgitation, and an atrial septal defect (ASD) versus a possible patent foramen ovale. All cases were noted to have connection from the persistent left superior vena cava to a coronary sinus which was dilated.

Two patients were noted to have arrhythmias. The first had premature atrial contractions (PACs) noted on fetal echocardiography with resolution in the postnatal period, while the second patient had SVT that developed postnatally, requiring amiodarone for rhythm control. There were several extracardiac findings noted as well. One patient was born with gastroschisis which was subsequently repaired. Two of our patients received genetic testing, the first for micrognathia which revealed a duplication/gain within 17p13.1, suggesting partial trisomy of this region, a variance of unknown significance. The other patient, who was noted to have supravalvular stenosis, received testing for Noonan Syndrome. Testing, however, was negative.

## Discussion

**Fig. 1:**
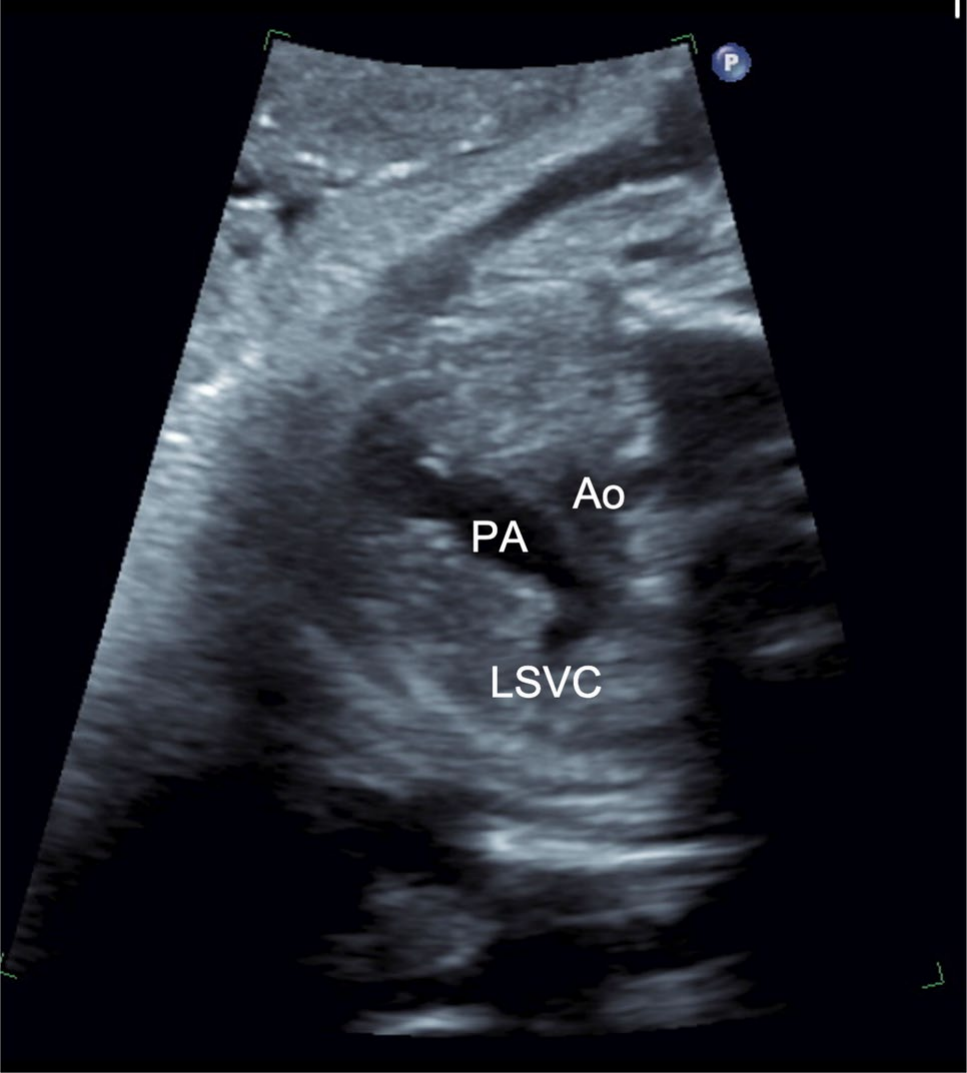
Abnormal three-vessel view on fetal echocardiogram, from left to right showing the PLSVC, Pulmonary Arteries, and Aorta

Of the detected cases of absent right with PSLVC in our series, the indication for fetal echocardiography ranged from cardiac anomalies, non-cardiac diseases, to maternal factors, warranting further work-up. The spectrum of indications emphasizes the importance of following a standardized protocol to ensure that subtle but clinically relevant diagnoses are not missed. Part of the standard examination of the fetal heart is the three-vessel view when scanning. In a normal vessel view, the arterial duct, aortic arch, and SVC will be seen from left to right. Instances in which a PLSVC is present, a fourth supernumerary vessel to the left of the pulmonary trunk and arterial duct will be seen [11]. Cases of absent right with PLSVC are more nuanced, as only three vessels will be seen but will be abnormally arranged, with PLSVC, arterial duct, and the aortic arch noted from left to right (Fig. 1) [8].

**Fig. 2:**
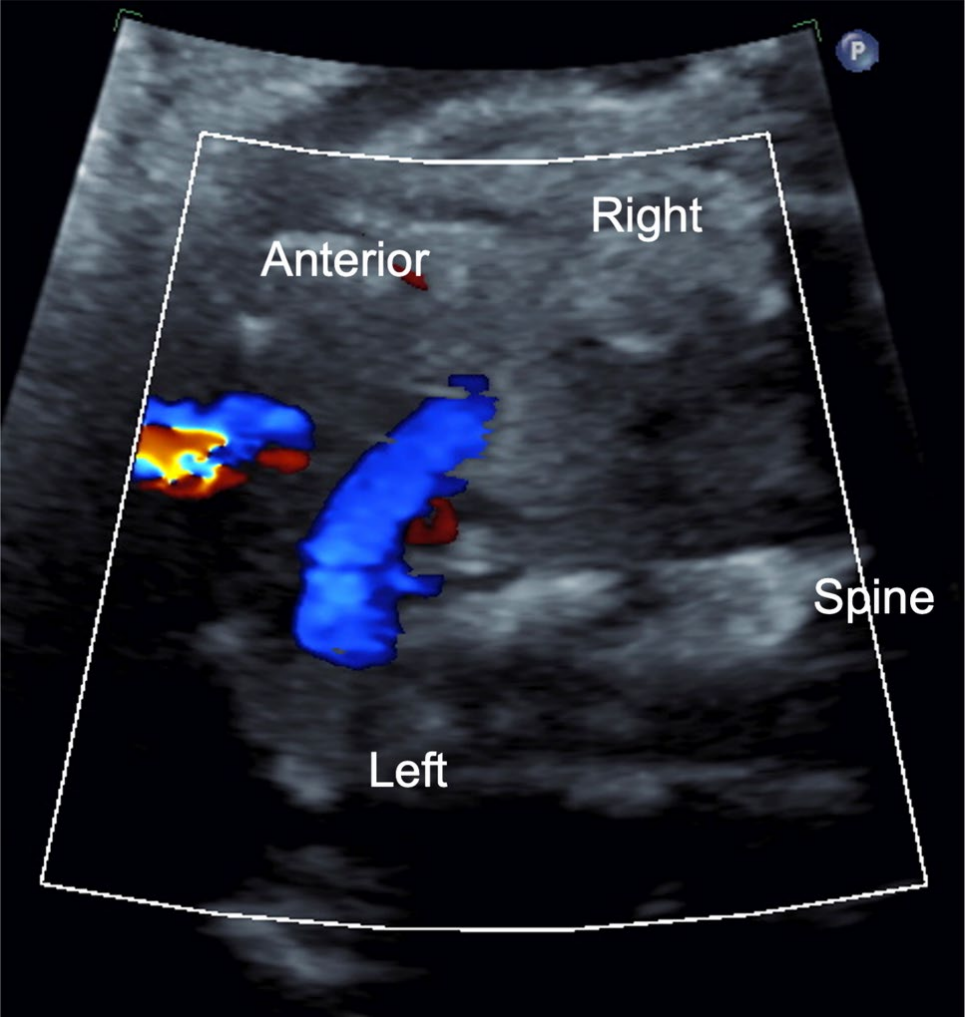
Flow reversal through the innominate vein, going from right to left into the PLSVC

**Fig. 3:**
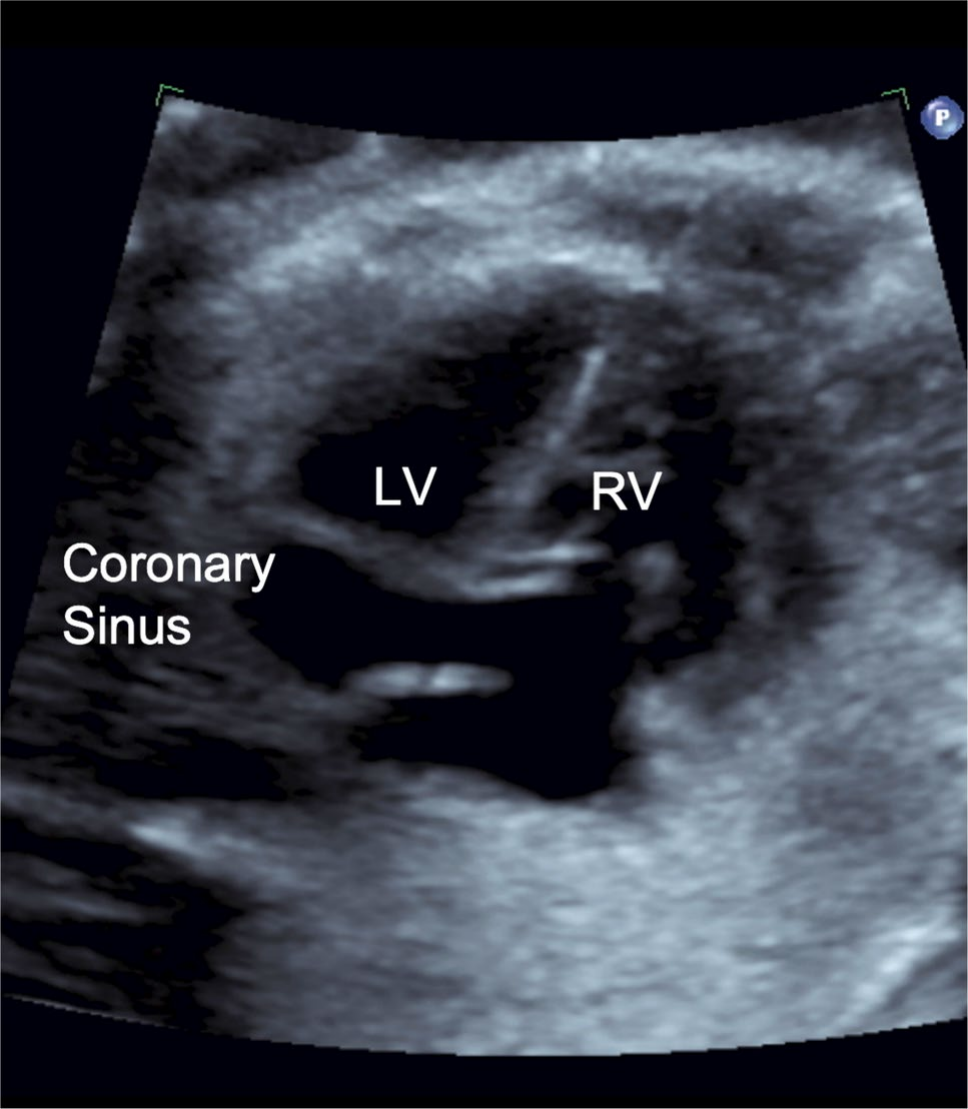
Image from a fetal echocardiogram showing a dilated coronary sinus a common finding in PLSVC

Additional consideration should be given to the innominate vein, as an IPLSVC will form if flow through the innominate vein is reversed, and the right cephalic region drains into the left anterior cardinal vein resulting in an atretic or absent RSVC (Fig. 2) [7]. Flow reversal in the innominate vein was seen in three of our patients. It is also imperative to evaluate the coronary sinus, as in all of our presented cases the coronary sinus was noted to be dilated and was the most consistently described feature consistent with absent right with PLSVC (Fig. 3) [5, 12].

Without other comorbidities, IPLSVC usually has no hemodynamic significance and requires no intervention but does have major clinical implications. Difficulties often related to implantation of a transvenous pacemaker, placement of a pulmonary artery catheter, cannulation of extracorporeal membrane oxygenation, and cannulation for bypass can all arise from the issues related to drainage into the coronary sinus [2, 3]. Coronary sinus dilation can lead to fragmentation and stretching of AV nodal tissue based on cell histology. Prior studies have shown persistent electrocardiographic anomalies in these patients, Bartam et al. showed 36% of patients with absent right and PLSVC had rhythm abnormalities, and Lenox et al. showed 80% of their patients having a shorter PR interval and a leftward frontal plane axis of the P wave, suggesting further evidence to support conduction changes associated with this diagnosis [2, 13]. However, most of these studies showed that the associated arrhythmias occurred in teenage years or into adulthood and, to our knowledge, little is known about any arrhythmias seen in the prenatal and immediate postnatal period.

Reviews of previous known cases of absent right with PLSVC showed additional cardiovascular anomalies in 46% of patients [2] These defects included atrial septal defects, coarctation of the aorta, endocardial cushion defects or tetralogy of Fallot. Cases of absent right with PLSVC have also been found in patients with chromosomal abnormalities including trisomy 21 and trisomy 18, with an incidence rate of 7% [2].

To our knowledge, no other case series have been reported with this magnitude of patients diagnosed with an absent right with PLSVC in the fetal period. While it was initially anticipated finding several patients with arch obstructions on known correlation and prenatal findings, ultimately there were none. However, there were other associated cardiac anomalies and complications, which merit further investigation. Due to the rarity of absent right with PLSVC, little is known about the spectrum and outcome of prenatally diagnosed cases. This patient population would benefit from a larger multicenter retrospective study to further characterize these rare anomalies and its associated complications.

## Data Availability

No datasets were generated or analysed during the current study.
